# Complementary description of *Colomerus novahebridensis* Keifer (Acari, Eriophyidae), with a discussion about the constitution of the genus and its economic importance, and a tentative key to *Colomerus* Newkirk & Keifer species

**DOI:** 10.3897/zookeys.434.7308

**Published:** 2014-08-14

**Authors:** Angsumarn Chandrapatya, Ploychompoo Konvipasruang, Carlos H. W. Flechtmann, Gilberto J. de Moraes

**Affiliations:** 1 Department of Entomology, Faculty of Agriculture, Kasetsart University, Chatuchak, Bangkok 10900, Thailand; 2 Plant Protection Research and Development Office, Department of Agriculture, Bangkok 10900, Thailand; 3 CNPq-Brazil Researcher, Departamento de Entomologia e Acarologia, Escola Superior de Agricultura “Luiz de Queiroz” (ESALQ) – Universidade de São Paulo, Piracicaba, SP, Brazil

**Keywords:** Taxonomy, Thailand, Eriophyoidea, Cecidophyinae

## Abstract

*Colomerus* Newkirk & Keifer, 1971 is an eriophyid genus described by Newkirk and Keifer about 43 years ago, that contains species from all continents, except Antarctica. They live mostly on dicotyledonous plants. *Colomerus novahebridensis* Keifer, 1977 was described from coconut (*Cocos nucifera* L., Arecaceae) fruits from Vanuatu. A description of a Thai population of this species is given in this paper. A revised characterization of *Colomerus* and a dichotomous key for the separation of the species presently considered to belong to this genus are provided, and a consideration about the importance of *Colomerus* species is presented.

## Introduction

*Colomerus* Newkirk & Keifer is a relatively small genus of eriophyid mites described about 43 years ago by Newkirk and Keifer (1971). The 27 species assigned to this genus have been described from all continents, except Antarctica.

All *Colomerus* species have been described from dicotyledonous plants, except *Colomerus novahebridensis* Keifer, described from coconut (*Cocos nucifera* L.; Arecaceae) (Keifer 1977). The latter species was originally collected from coconut fruits in Saraoutou, Vanuatu (mentioned in the original description as New Hebrides Islands, the former name of that archipelago). Specimens identified in the present paper as *Colomerus novahebridensis* were found a few years ago by the authors of this paper while unsuccessfully searching for the possible presence of an economically important eriophyid species, *Aceria guerreronis* Keifer, 1965, on coconut in Thailand.

The objective of this paper is to present a morphological description of that Thai population (based on adult females and males), to discuss the constitution of the genus, to provide a tentative dichotomous key to *Colomerus* species worldwide and to summarize the economic importance of this genus.

## Materials and methods

Specimens used for the complementary description of *Colomerus novahebridensis* were collected in different coconut fields in the central and southern regions of Thailand. Coconut fruits with symptoms of eriophyid attack similar to that of *Aceria guerreronis* (whitish to brownish triangular scars starting at the edge of the bracts and progressively enlarging with fruit growth) were collected and taken to the laboratory for examination. The bracts were removed and their undersurfaces as well as the surface of the fruits covered by them were examined, collecting all eriophyid mites found.

The mites were mounted in modified Berlese medium (Amrine and Manson 1996) for later examination under an Olympus BX 43 microscope with phase contrast. Structures relevant for taxonomic purposes were measured using a graded eyepiece and illustrated using a camera lucida attached to the microscope. Both photographs and scanning electron micrographs of specimens from the collection of H.H. Keifer (ARS, USDA, Beltsville, Maryland, USA), were taken by Philipp Chetverikov (Biological Research Institute, St. Petersburg State University, Old Peterhof, Russia), who kindly made them available to us for comparison with specimens we collected (these were not included in this publication). Notes on the bag containing the dry specimens mounted by P. Chetverikov read “ex. coconut cap, *Cocos nucifera*; Thailand, at Los Angeles; July 8, 1975”, probably referring to specimens intercepted at Los Angeles, California, USA, from coconuts imported from Thailand.

All terminology and measurements follow Lindquist (1996) and de Lillo et al. (2010). The measurements are given in micrometers. Opisthosomal dorsal annuli count starts at the posterior shield margin; ventral annuli count starts from the first lateral annulus at the lateral prodorsal shield margin; the length of each leg is measured from the trochanter base to the tip of tarsus, excluding empodium. All specimens examined are deposited in the Insect Museum of Department of Entomology, Kasetsart University, Bangkok, Thailand, and Museum of Department of Agriculture, Ministry of Agriculture and Cooperatives, Bangkok, Thailand.

The revised characterization of the genus and the dichotomous key were prepared by examining the original descriptions of each species, except for *Colomerus novahebridensis*, collected in this work, *Colomerus bucidae* (Nalepa), whose characteristics were taken from Flechtmann et al. (2000) and from our examination of specimens collected in the Dominican Republic by L. Sánchez-Ramirez (unpublished), and for *Colomerus vitis* (Pagenstecher), whose characteristics were taken from an examination of specimens collected from grapevine buds in Candiota and Bento Gonçalves, both in the state of Rio Grande do Sul, Brazil by N.J. Ferla. The key should be considered as tentative, because it was not possible in the scope of this work to study the actual type specimens of the species involved. Given the limited information provided in the description of some of the species, some of the characters used in the key cannot be considered as robust as desirable. Thus, its use should always be associated with complementary examination of the original description of the species thus determined. The species considered in this study are those listed in Amrine and Stasny (1994), complemented by the unpublished computerized database of world eriophyoid species compiled by Amrine and de Lillo (pers. comm.).

## Results and discussion

### Colomerus novahebridensis

Taxon classificationAnimaliaProstigmataEriophyidae

Keifer

Colomerus novahebridensis Keifer, 1977: 23–24

#### Diagnosis.

Frontal lobe of prodorsal shield rounded, broad-based, short; with parallel microtuberculate lines around lateral margin of ocellar gibbosities; median and admedian lines between anterior shield margin and region slightly anterior to shield center usually broken (indistinct in some specimen), and then continuous to posterior shield margin (broken in some specimens); with several incomplete submedian lines; empodia entire, 5-rayed; opisthosoma with 67–85 microtuberculate annuli; coverflap with longitudinal ridges arranged in two transverse rows. Genital apodeme usually visible as a narrow dark band in ventral view, but sometimes appearing to constitute a pair of subtriangular structures, depending on the position of the focus; spermathecal apparatus moderate distance from apodeme; with 4 coxigenital semiannuli anterior to coverflap, with genital opening somewhat appressed to coxisternum II.

#### Description.

**Figure 1.**
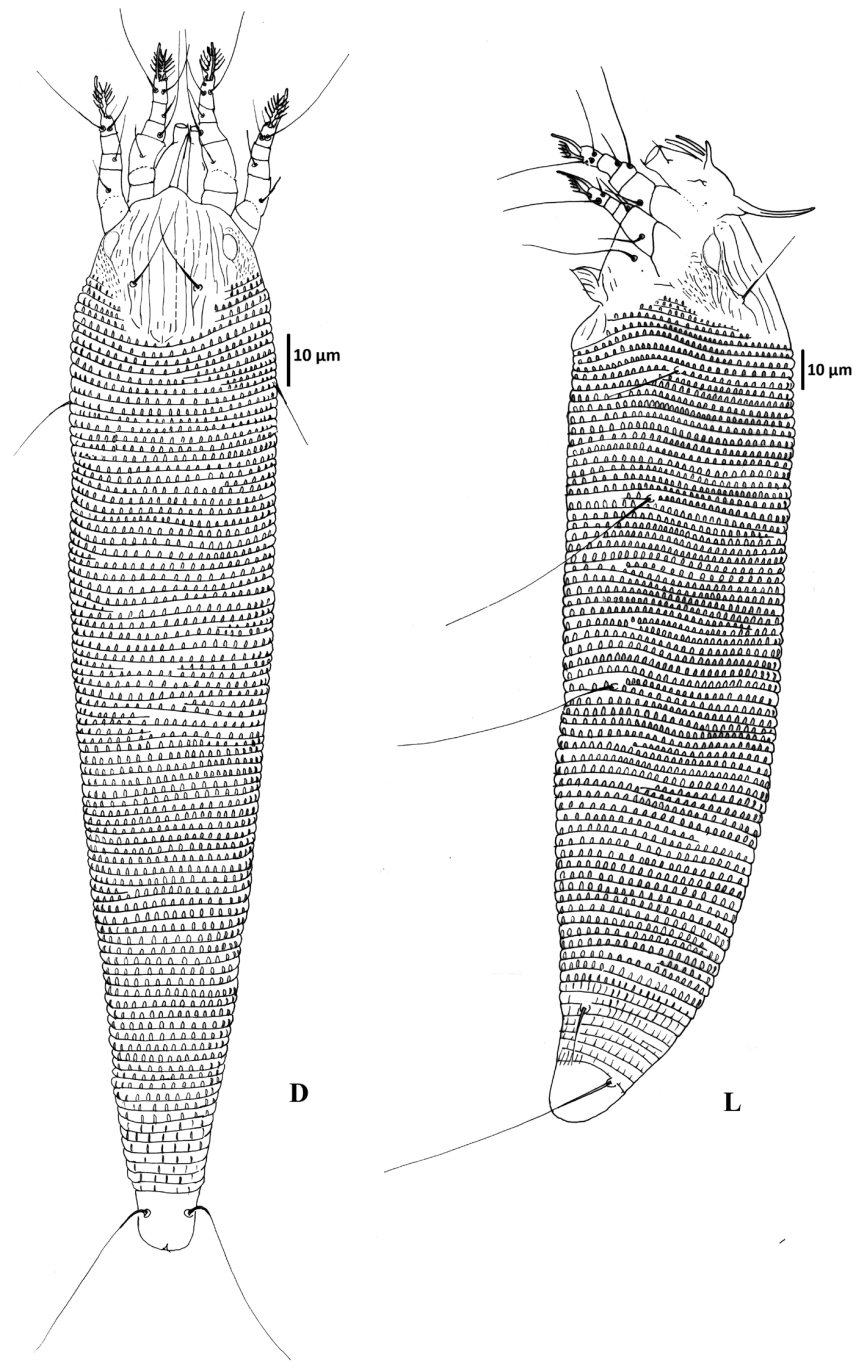
*Colomerus novahebridensis* Keifer. Female: **D** = dorsal view, **L** = lateral view. Specimens collected in Thailand.

**Figure 2.**
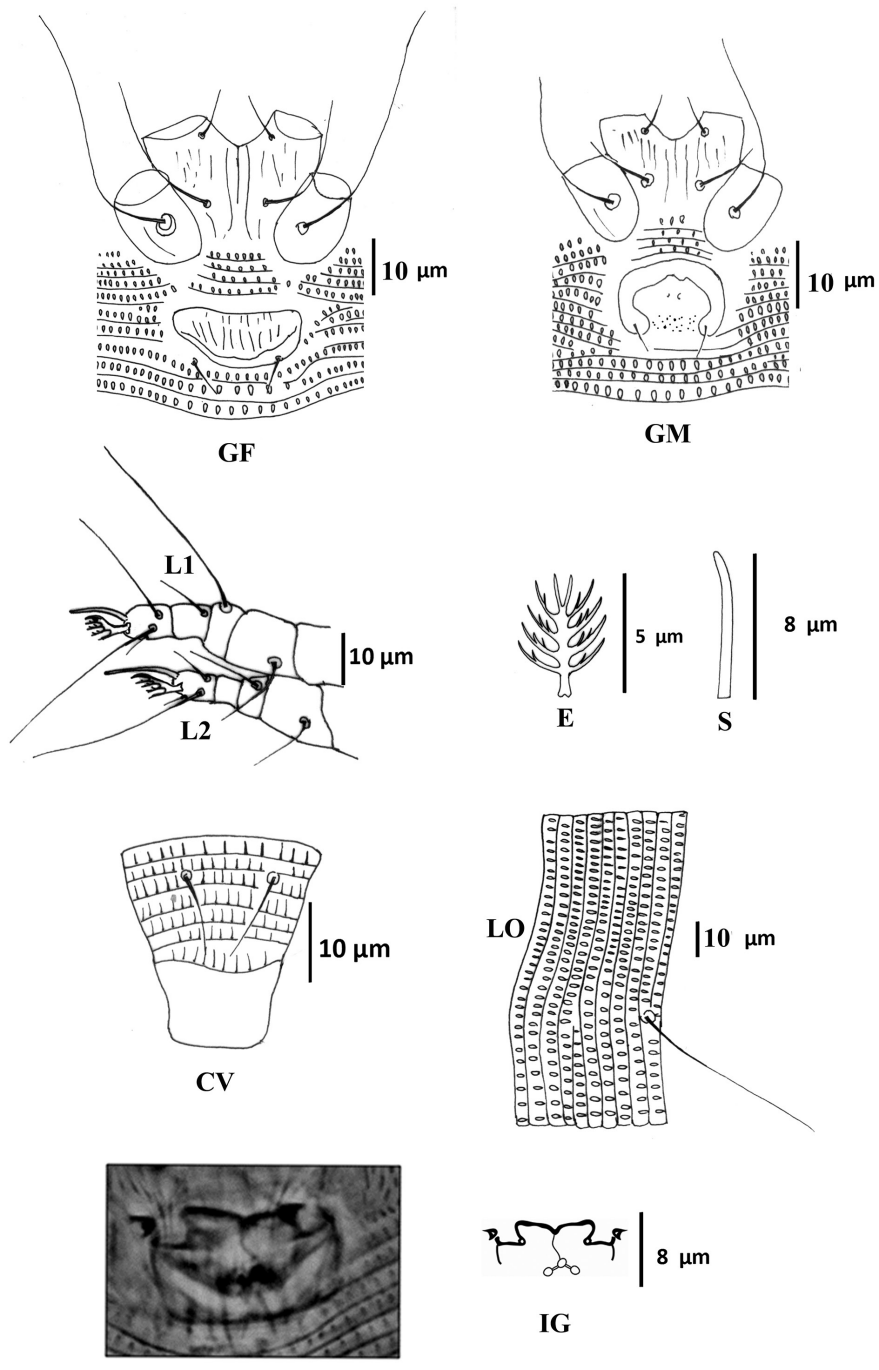
*Colomerus novahebridensis* Keifer. Female: **CV** ventral view of caudal region **E** empodium **GF** external female genitalia **IG** internal genitalia **L1** leg I **L2** leg II **LO** lateral opisthosoma **S** solenidion. Male: **GM** external male genitalia. Specimens collected in Thailand.

**Figure 3.**
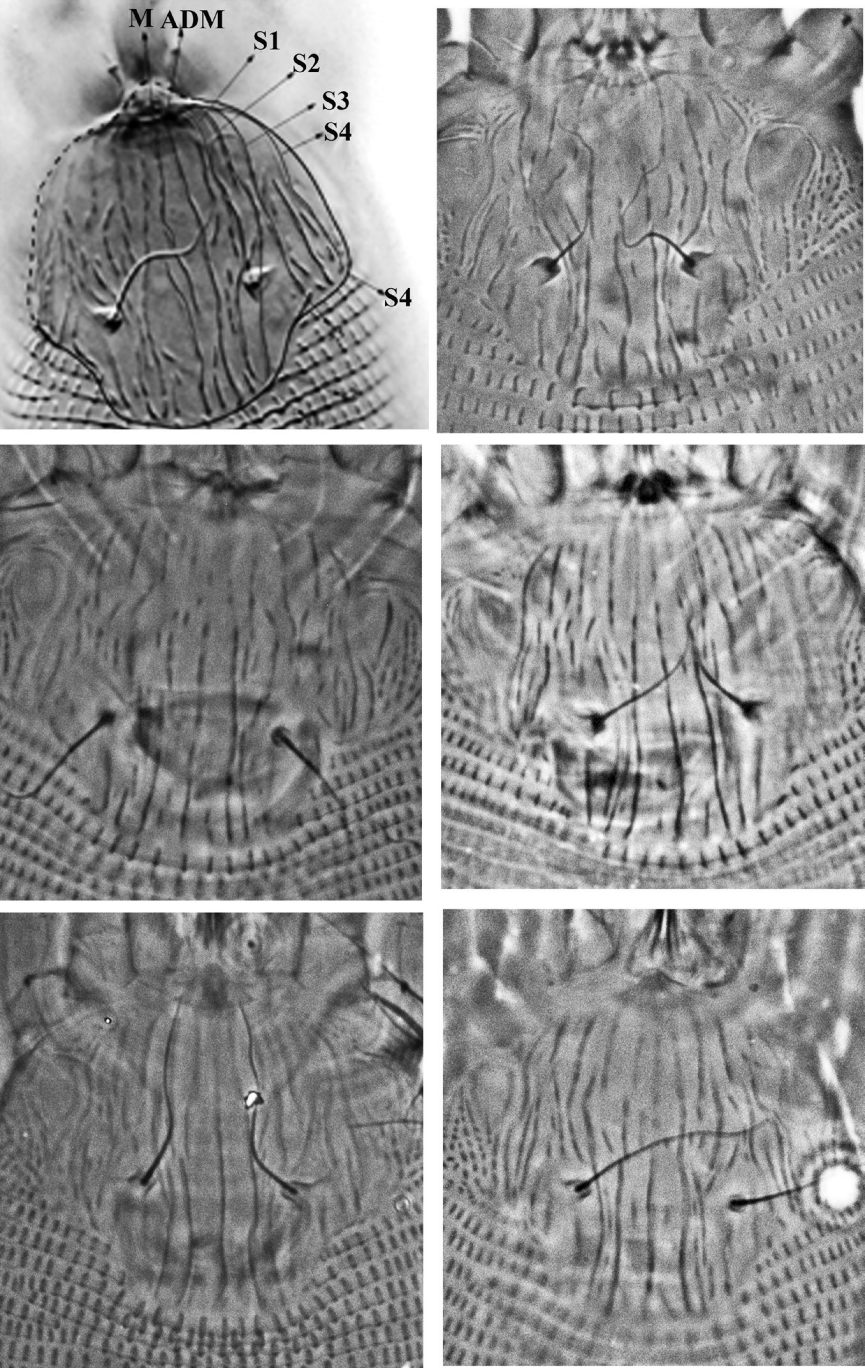
Variation of prodorsal shield sculpture of *Colomerus novahebridensis* Keifer. The top left figure highlights the prodorsal shield lines: from center to lateral margin, lines running from anterior to posterior margin are interpreted as median (**M**), admedian (**ADM**) and submedian lines (**S1–S4**). Specimens collected in Thailand.

Female (Figs 1–3) (n = 9). Body wormlike, 187–238, 41-47 wide, 47–49 thick, whitish. **Gnathosoma** (Fig. 1): 16–18, projecting slightly downwards, pedipalp coxal seta (*ep*) 2–3, dorsal pedipalp genual seta (*d*) 5–7, subapical pedipalp tarsal seta (*v*) 2, cheliceral stylets 14–21. **Prodorsal shield** (Figs 1–3): 28–41, 34–41 wide, semi-oval; prodorsal shield frontal lobe rounded, broad-based, short, 2–3; posterior shield margin convex, interrupting first 4–5 dorsal annuli. Prodorsal shield design with parallel microtuberculate lines around lateral margin of ocellar gibbosities. Line pattern variable (Fig. 3); median and admedian lines usually broken (indistinct in some specimens) between anterior shield margin and region slightly anterior to shield center and then continuous to posterior shield margin (broken in some specimens); some specimens with 1–2 short lines between median and admedian lines near posterior margin of prodorsal shield. Submedian lines variously broken, typically in four pairs running from anterior to posterior margins and four incomplete submedian lines running from anterior to posterior margin; 2 – 3 submedian lines posteriad or mesad of scapular tubercles; ocellar gibbosities prominent. Scapular tubercles situated 7–11 ahead of posterior shield margin, plicate, 12–14 apart, scapular setae (*sc*) 16–19, directed upward or forward. **Coxigenital region:** with 4 coxigenital semiannuli, microtuberculate. **Coxisternal figs** (Fig. 2 GF): coxisternum I with several longitudinal lines, coxisternum II smooth, anterior seta on coxisternum I (*1b*) 5–6, 9–10 apart; proximal seta on coxisternum I (*1a*) 15–22, 8–9 apart; proximal seta on coxisternum II (*2a*) 28–39, 19–21 apart; tubercles of *1b* and *1a* 8–10 apart. Internal coxisternal apodeme 9–12. **Legs** (Fig. 2 L1, L2, E, S): with all usual setae. Leg I 23–29, femur 8–10, ventral basifemoral seta (*bv*) 6–8; genu 4–5, antaxial genual seta (*l"*) 17–22; tibia 4–5, paraxial tibial seta (*l*') 4–6; tarsus 5–6, antaxial fastigial tarsal seta (*ft"*) 16–18, paraxial fastigial tarsal seta (*ft*') 10–16, paraxial unguinal tarsal seta (*u*') 3, tarsal empodium 6–8, entire, 5-rayed, tarsal solenidion (*ω*) 6–10, slightly curved, blunt. Leg II 22–26, femur 6–10, ventral basifemoral seta (*bv*) 5; genu 3–4, antaxial genual seta (*l"*) 5–8; tibia 3–4; tarsus 4–6, antaxial fastigial tarsal seta (*ft"*) 18–23, paraxial fastigial tarsal seta (*ft*') 4–6, paraxial unguinal tarsal seta (*u*') 2–5, tarsal empodium 6–8, entire, 5-rayed, tarsal solenidion (*ω*) 8–9, slightly curved, blunt. **Opisthosoma** (Fig. 1D and L, Fig. 2 ES, CV): dorsum evenly rounded, dorsal annuli 67–83, ventral annuli 71–85, both with elongate, oval microtubercles situated on or near posterior margin of each annulus. Microtubercles more elongate on the last 5–7 ventral annuli and slightly longer, sparser on the last 7-8 dorsal annuli. Seta *c2* 17–22, 39–46 apart, on ventral annulus 10–12; seta *d* 43–50, 33–39 apart, on ventral annulus 21–27; seta *e* 44–64, 19–24 apart, on ventral annulus 37–49; seta *f* 10–13, 11–13 apart, on ventral annulus 66–80 or annulus 5th from the rear. Seta *h_1_* absent, *h_2_* 38–53. **Female genitalia** (Fig. 2 GF, IG): 8–9, 18–20 wide, coverflap with 8–12 longitudinal ridges in each of two transverse rows, setae *3a* 4–6, 11–13 apart. Internal genital apodemes usually visible as a narrow dark band in ventral view (Fig. 2 IG), but sometimes appearing to constitute a pair of subtriangular structures (Fig. 2 IG), depending on the position of the focus; spermathecal apparatus at moderate distance from apodeme.

**Male** (Fig. 2 GM) (n =3): smaller than female, 150–170, 40–48 wide, 44 thick. **Gnathosoma:** 16–18; pedipalp coxal seta (*ep*) 2, dorsal pedipalp genual seta (*d*) 5–6, subapical pedipalp tarsal seta (*v*) 2, cheliceral stylets 15–17. **Prodorsal shield:** 30–34, 34–35 wide, prodorsal shield frontal lobe rounded, broad-based, 2–3, shield design similar to that of the female; ocellar gibbosities prominent. Scapular tubercles situated 6–8 ahead of posterior shield margin, plicate, 14–15 apart; scapular setae (*sc*) 10–12, directed upward or forward. **Coxigenital region:** with 4 coxigenital semiannuli, microtuberculate. **Coxisternal figs** (Fig. 2 GM): coxisternum I with several longitudinal lines, coxisternum II smooth, anterior seta on coxisternum I (*1b*) 5–6, 9–10 apart; proximal seta on coxisternum I (*1a*) 14–16, 8–9 apart; proximal seta on coxisternum II (*2a*) 24–26, 18–19 apart, tubercles *1b* and *1a* 8 apart. Internal coxisternal apodeme 8–11. **Legs:** with usual setae. Leg I 21–24, femur 7–8, ventral basifemoral seta (*bv*) 5–8; genu 4–5, antaxial genual seta (*l"*) 15–24; tibia 4–5, paraxial tibial seta (*l*') 4–5; tarsus 4–5, antaxial fastigial tarsal seta (*ft"*) 14–18, paraxial fastigial tarsal seta (*ft*') 12–13, paraxial unguinal tarsal seta (*u*') 2–3, tarsal empodium 5–6, entire, 5-rayed, tarsal solenidion (*ω*) 6–8, slightly curved, blunt. Leg II 18–20, femur 7, ventral basifemoral seta (*bv*) 6–7; genu 3, antaxial genual seta (*l"*) 4–5; tibia 3; tarsus 5–6, antaxial fastigial tarsal seta (*ft"*) 16–19, paraxial fastigial tarsal seta (*ft*') 4–6, paraxial unguinal tarsal seta (*u*') 2–3, tarsal empodium 5–6, entire, 5-rayed, tarsal solenidion (*ω*) 10, slightly curved, blunt. **Opisthosoma:** dorsum evenly rounded, dorsal annuli 59–63 and ventral annuli 63–66. Seta *c2* 16, 40–47 apart, on annulus 9–10; seta *d* 30–32, 26–29 apart, on annulus 19–20; seta *e* 40–45, 16–21 apart, on annulus 34–36; seta *f* 10, 12–13 apart, on annulus 56–61 or annulus 5th from the rear. Seta *h_1_* absent, *h_2_* 28–35. **Male genitalia** (Fig. 2 GM) 10–14, 18–19 wide, seta *3a* 4–6, 10–12 apart.

#### Material examined.

12 adult females and 5 adult males on 14 slides labeled # 2874, from Mueang Samut Songkhram District, Samut Songkhram Province, 13°24.834'N, 100°0.198'E, 14-II-2011, coll. P. Vichitbandha and G. J. de Moraes; 5 adult females on 5 slides labeled # 2875, from Chumporn Province, 10°15.2'N, 99°5.7'E, 14-II- 2011, coll. P. Vichitbandha and G. J. de Moraes; 3 adult females on 2 slides labeled # 2876, Ban Phaeo District, Samut Sakhon Province,13°35.433'N, 100°6.466'E, 15-II-2011, coll. P. Vichitbandha and G. J. de Moraes; 7 adult females on 7 slides labeled # 2878 and 5 adult females and 1 adult male on 6 slides labeled # 2879, Kanchanadit District, Surat Thani Province, 9°9.933'N, 99°28.266'E, 15-II-2011, coll. P. Vichitbandha and G. J. de Moraes; 8 adult females on 7 slides labeled # 2883, Kanchanadit District, Surat Thani Province, 9°9.933'N, 99°28.266'E, 23-II-2011, coll. Yingniyom Riyaphan; 3 adult females, 1 adult male and 1 nymph on 5 slides labeled # 2911, Kanchanadit District, Surat Thani Province, 9°9.933'N, 99°28.266'E, 12-IX-2011, coll. Yingniyom Riyaphan; 72 adult females, 6 adult males and 5 nymphs on 23 slides labeled # 2912, Kanchanadit District, SuratThani Province, 9°9.933'N, 99°28.266'E, 28 IX 2011, coll. Yingniyom Riyaphan.

#### Host.

Coconut (*Cocos nucifera* L. var. *nucifera*, Ma phrao; Arecaceae)

#### Relation to host.

All specimens were collected from under the bracts of coconut fruit, causing usually the appearance of scanty triangular brown patches of damaged tissue on the fruit surface next to the bracts under which the colonies of the mites developed. In a few occasions damage was slightly more extensive, and the mite apparently caused premature fruit drop.

### Remarks

The morphological characteristics described generally fit the original description of the species, which was much less detailed. Slight differences, subsequently referred to, are considered to represent intraspecific variations. In the original description, admedian lines were mentioned as being complete, which was not the case with the specimens collected in this study. The illustration provided in the original description of the species indicates the presence of a few more submedian lines than observed in the specimens from Thailand. The original description mentioned frontal lobe of prodorsal shield to be truncate. The illustration of prodorsal shield design in the original description shows six partial rings antero-laterally, which is not seen in our specimens; internal coxisternal apodeme is also present in some Thai specimens, but it is not shown in the original description.

### Revised characterization of *Colomerus*

**Type species:** *Eriophyes gardeniella* Keifer, by original designation.

As stated by Newkirk and Keifer (1971), this genus was erected to include species until then considered to belong to *Eriophyes* von Siebold (subfamily Eriophyinae), but that had genitalia and coxal structures typical for Cecidophyinae, namely *Colomerus gardeniella* (Keifer), *Colomerus holodisci* (Keifer) and *Colomerus vitis*.

Keifer (1977) assumed the following characteristics as essential for the placement of species in this genus: a) genital opening somewhat appressed to hind coxae [in our concept, with a maximum of 4 coxigenital semiannuli anterior to coverflap]; b) genital apodemes appearing narrow [mentioned as “always shortened in ventral view, but somewhat variable” in the original description and mentioned as shortened by Keifer (1977)] in ventral view; c) scapular seta [named dorsal seta by Keifer (1977)] directed diagonally ahead or straight ahead; d) genital coverflap with longitudinal ridges arranged in two uneven transverse rows.

An evaluation of the species assigned to this genus leads to the conclusion that the first of those characteristics (position of genital opening) holds true for all of them. In relation to the second characteristic, the majority of the species placed in this genus has been mentioned to have narrow genital apodemes. However, nothing has been mentioned in the literature about the shape of the genital apodemes of *Colomerus oculivitis* (Attiah 1967). In a personal communication to the authors of the present publication (January 2014), C. Craemer kindly informed that in her evaluation of the specimens of the *Colomerus vitis* – *Colomerus oculivitis* complex (see Craemer and Saccaggi 2013), some specimens showed the typical narrow genital apodemes, whereas others showed genital apodemes as a pair of twisted leaf-like structures, similarly to what was observed in the present study for the specimens from Thailand identified as *Colomerus novahebridensis*. Subtriangular apodemes were observed in specimens identified as *Colomerus vitis* from southern Brazil.

Available illustrations of *Colomerus codiaeum* Keifer, 1979 and *Colomerus trichodesmae* Chakrabarti & Pandit, 1997 do not show the typical (narrow) apodemes illustrated by Keifer for the type species of the genus. The inclusion of *Colomerus codiaeum* in this genus is intriguing, given that it was described by Keifer, just two years after he published the items he considered essential for *Colomerus* species. Did he make a mistake in accepting that species as *Colomerus*? Did he then decided that species with different shape of genital apodeme could still be included in that genus, even without explicitly saying so, as could be assumed from his statement in the original description “always shortened in ventral view, but somewhat variable?”. In this publication, we will accept the second option to be the case. This statement by Keifer reflects the assumed variability of the observed shape of these internal structures viewed under phase or interference contrast microscopy. Attempts to determine the real format of these structures could greatly benefit from observations under confocal microscopy, as used by Chetverikov (2014) for the study of other eriophyoids.

Nothing has been reported about the shape of the genital apodemes for the following species transferred to or originally described in *Colomerus*: *Colomerus bucidae* (Nalepa, 1904), *Colomerus lepidaturi* (Farkas, 1960), *Colomerus pruni* Kuang & Luo, 2005 (in Kuang et al. 2005), *Colomerus robaticus* Xue, Sadegui & Hong, 2012 and *Colomerus spathodeae* (Carmona, 1967). Examination of the specimens redescribed by Flechtmann et al. (2000) and of the specimens from the Dominican Republic did not allow the verification of the shape of the genital apodeme.

An evaluation of the species referred to *Colomerus* suggested that it is not convenient to consider the orientation of the scapular seta as characteristic for species to be placed in this genus, given that it may vary when a specimen is slide mounted, although the species referred to this genus in the literature have been rarely mentioned or illustrated as having the scapular seta directed backward [only some *Colomerus bucidae*, according to Flechtmann et al. (2000) and according to our examination of specimens from the Dominican Republic]. Also, it is not considered essential that the ridges of the coverflap be arranged in two uneven transverse rows, given that a continuous variation was observed (as subsequently detailed) from one to two transverse rows in species that otherwise resemble other species placed in this genus, as characterized later in this paper.

In the original description, *Colomerus pruni* has been mentioned to have *h1* [rarely reported in other *Colomerus* (see characterization below)]; this species as well as *Colomerus robaticus* have non-microtuberculate dorsal annuli and genital coverflap without ridges. Thus, they are not considered for the new characterization subsequently proposed for this genus, as they probably belong to a different genus (genera). Conversely, *Colomerus trichodesmae*, *Colomerus bucidae*, *Colomerus lepidaturi* and *Colomerus spathodeae* are provisionally retained in *Colomerus*, despite the reportedly non-typical genital apodeme of the first species or the absence of information about the shape of genital apodemes for the others.

A revised characterization of *Colomerus* could be stated as follows.

**Idiosoma:** wormlike, with opisthosomal annuli subequal dorsoventrally and microtuberculate; in some species smooth on the few posterior-most opisthosomal annuli (in the original description of *Colomerus gardeniella*, type species of the genus, microtubercles very faint or absent dorsally on the six posterior-most dorsal annuli); opisthosomal setae *h1* absent [except, either reduced or completely absent in *Colomerus neopiperis* (Wilson, 1970), according to Wilson (1970) and usually absent in *Colomerus nudi* Manson, 1984, according to Manson (1984)]; mentioned and illustrated as present in the original description of *Eriophyes buceras* Cromroy, 1958, but not seen in specimens reported by Flechtmann et al. (2000) as *Colomerus bucidae* (Nalepa 1904), considered in that paper to be the senior synonym of the former species. Seta *h1* was also absent in the specimens of this species collected in the Dominican Republic and examined in this study.

**Prodorsal shield:** anterior lobe varying from indistinguishable to distinctly triangular or round and broad-based [absent according to original description of the genus]; scapular tubercles positioned variably from very near posterior shield margin to well anterior to posterior shield margin [slightly anterior to posterior shield margin according to original description of the genus, directing scapular setae diagonally forward or straight ahead (occasionally backward or laterally) [directing setae up and ahead in some degree according to original description of the genus]; gnathosoma short.

**Legs:** coxae I widely separate, with moderate or short internal coxisternal apodeme (in some species, anterior coxisternal regions totally separated and internal coxisternal apodeme not seen); legs with all usual setae, empodia entire, 4–6 rayed [only species with 5 rayed included in the original description].

**Female genitalia:** genital opening somewhat appressed to coxisternum II (4 coxigenital semiannuli anterior to genital coverflap); coverflap with longitudinal ridges distinctly arranged in one or two transverse rows, or with some (shorter) ridges in two rows and some (longer) ridges running along most of the length of genital coverflap, constituting a single row [arranged in uneven double rows according to original description of the genus]; genital apodemes usually visible as a narrow dark band in ventral view, but sometimes appearing to constitute a pair of subtriangular structures, depending on the position of the focus.

### Key for the separation of the world *Colomerus* species (based on adult protogyne females)

*Eriophyes buceras* Trotter, 1929 should not be confused with *Eriophyes buceras* Cromroy, 1958. As there is no satisfactory description of the first of these species, a confirmation of its generic placement cannot be done. The second species was considered by Flechtmann et al. (2000) to be a junior synonym of *Colomerus bucidae*. Some differences are observed between the redescription of *Colomerus bucidae* given by Flechtmann et al. (2000) and the original description of *Eriophyes buceras* Cromroy, including the absence of seta *h1* in the specimens reported by Flechtmann et al. (2000) (also in the types of *Colomerus bucidae*, as apparently mentioned in the original description: “s.a.fehlen”) and the presence in the types of *Eriophyes buceras* Cromroy. Carlos Flechtmann considers however that those differences could correspond to misinterpretation of structures when Cromroy described his specimens. According to Cromroy (1958), *Colomerus buceras* causes 4 distinct types of injury to its host, namely a deformation of fruits, erinea on the leaves, and 2 different types of galls. However, it seems that these symptoms are not the same as those reported by Trotter (1929) for the species he had described as *Eriophyes buceras*, mentioned to consist of distinctive elongated, slender, hollow, horn-shaped flower outgrowths, reaching about 19 cm in length (very long, thin galls produced instead of the normal fruit measuring only 5–6 mm) and about 2–4 mm thick; some galls may develop into witches' brooms type of deformation. In the original description of *Colomerus bucidae*, symptoms are mentioned as erineum-like structures in depressions of the undersurface of the leaves. Thus, these differences, although caused to the same host plant (*Terminalia buceras*, senior synonym of *Bucida bucera* and *Buchenavia buceras*), suggest *Eriophyes buceras* Trotter to be different from *Colomerus bucidae* and *Colomerus buceras* Cromroy. In the original description of *Colomerus buceras* Trotter, the author mentioned it to be similar to *Colomerus bucidae*. *Eriophyes buceras* Trotter needs to be redescribed.

In a recent publication, Craemer and Saccaggi (2013) reported an extensive evaluation of eriophyid mites intercepted on grape berries and grapevine budwood imported to South Africa from various countries. The authors reported their uncertainty in relation to the reliable separation of *Colomerus vitis* and *Colomerus oculivitis*, given the high variability of characters considered important in the characterization of those species, observed in their examination of specimens and available redescriptions of *Colomerus vitis*. They reported that the only discrete and unambiguous distinguishing character was the number of empodial rays (5 in *Colomerus vitis* and 6 in *Colomerus oculivitis*), with a possible additional difference related to the shape and density of opisthosomal tubercles (rounded and more widely spaced in *Colomerus vitis* as opposed to elongate and closer together in *Colomerus oculivitis*). Despite those cited differences, the authors claimed that *Colomerus* mites from grapevine worldwide could not be accurately identified to species, given the possible (but not detected) variation in the number of empodial rays in those species. Regardless of that uncertainty, those species are placed separately in the key subsequently provided in this publication.

*Eriophyes vitigineusgemmae* Mal’chenkova, 1970 may also belong to *Colomerus*. However it is not included in the subsequent key because, according to the original description, its coverflap does not seem appressed to coxisternum II and because nothing has been mentioned about its genital apodemes.

*Colomerus pruni* and *Colomerus robaticus* are also not included in the key because they probably belong to a different genus (genera), as previously discussed in this publication.

**Table:** 

1	Without evident ocellar gibbosities; empodia 4-rayed	2
1’	With or without evident ocellar gibbosities; empodia 5- or 6-rayed	3
2	Prodorsal shield without frontal lobe; region between admedian lines with many short lines; on *Trichodesma khasianum*	*Colomerus trichodesmae* Chakrabarti & Pandit, 1997
2’	Prodorsal shield with frontal lobe; region between admedian lines with few short lines; on *Gardenia volkensii* subsp. *volkensii*	*Colomerus volkensiae* Meyer & Ueckermann, 1990
3	With evident ocellar gibbosities; empodia 6-rayed; all opisthosomal annuli microtuberculate	4
3’	With or without ocellar gibbosities; empodia 5-rayed; posterior-most opisthosomal dorsal annuli with or without microtubercles	6
4	Opisthosomal seta *e* slightly over half as long as opisthosomal seta *d* and about as long as opisthosomal seta *f*; on *Woodfordia floribunda*	*Colomerus woodfordis* Ghosh & Chakrabarti, 1989
4’	Opisthosomal seta *e* at least 1.2 times as long as opisthosomal seta *d* and at least 3.5 times as long as opisthosomal seta *f*	5
5	Scapular seta *sc* 21 µm; opisthosomal seta *d* 36 µm; opisthosoma with 70 annuli; microtubercles very narrow (linear); on *Vitis vinifera*	*Colomerus oculivitis* (Attiah, 1967)
5’	Scapular seta *sc* 10 µm; opisthosomal seta *d* 25 µm; opisthosoma with 55–62 annuli; microtubercles ovoid to rounded; on *Piper jaliscanum*	*Colomerus neopiperis* (Wilson, 1970)
6	Prodorsal shield smooth, except for few curved broken bases of admedian lines restricted to region between scapular tubercles and a tiny remnant of median line; without evident ocellar gibbosities; most posterior dorsal opisthosomal annuli without microtubercles; on *Baloghia inophylla* (G.Forst.) P.S. Green (mentioned as *Codiaeum inophyllum*, junior synonym)	*Colomerus codiaeum* Keifer, 1979
6’	Prodorsal shield with more extensive lines; with or without evident ocellar gibbosities; most posterior dorsal opisthosomal annuli with or without microtubercles; on other hosts	7
7	Median line on prodorsal shield only distinguishable posteriorly, joined by broken arched lines to admedian lines, so as to form a pair of roundish cells at the base of the admedian lines; genital coverflap with longitudinal ridges arranged in two distinct transverse rows, those of the anterior row much shorter, fine and less evident than those of the posterior row; on *Gardenia jasminoides*	*Colomerus gardeniella* (Keifer, 1964)
7’	Median line on prodorsal shield not joined by broken arched lines to admedian lines; longitudinal ridges of genital coverflap not characteristically arranged in two transverse rows or, if so, then anterior row not composed of distinctly shorter, fine and less evident ridges than those of the posterior row	8
8	Prodorsal shield with frontal lobe (sometimes barely distinguishable)	9
8’	Prodorsal shield without frontal lobe	19
9	Prodorsal shield with lateral granulation; without evident ocellar gibbosities	10
9’	Prodorsal shield without lateral granulation; with or without evident ocellar gibbosities	11
10	Opisthosomal setae *d* and *e* 30 and 8 µm, respectively; opisthosoma with 48 microtuberculate annuli; on *Holodiscus microphyllus*	*Colomerus holodisci* (Keifer, 1970)
10’	Opisthosomal setae *d* and *e* 18–25 and 18–30 µm, respectively; opisthosoma with 55–70 annuli; microtubercles missing on posterior 6–7 dorsal annuli; on *Phebalium nudum*	*Colomerus nudi* Manson, 1984
11	Opisthosoma with 60–85 annuli	12
11’	Opisthosoma with less than 60 annuli (except *Colomerus coplus*, with 53–63)	14
12	With evident ocellar gibbosities; with 67–85 microtuberculate annuli; on *Cocos nucifera*	*Colomerus novahebridensis* Keifer, 1977
12’	Without evident ocellar gibbosities; with 61–75 annuli, all microtuberculate or posterior ten dorsal annuli with few microtubercles	13
13	Opisthosoma with 61–68 annuli; posterior 10 dorsal annuli with few microtubercles; on *Tricalysia junodii* var. *junodii* and *Sericanthe andongensis*	*Colomerus tricaseri* Meyer & Ueckermann, 1990
13’	Opisthosoma with 75 microtuberculate annuli; on *Diospyros mespiliformis*	*Colomerus mespiliformae* Meyer & Ueckermann, 1990
14	Admedian lines on prodorsal shield well defined and complete; ocellar gibbosities absent; all opisthosomal annuli microtuberculate	15
14’	Admedian lines on prodorsal shield generally not well defined (or broken), may be distinct on posterior half of prodorsal shield; microtubercles may be absent on posterior opisthosomal dorsal annuli	16
15	Median line totally distinct; opisthosoma with 53–63 microtuberculate annuli; opisthosomal setae *d* and *e* 19–24 and 14–26 µm, respectively; on *Melicope simplex* A. Cunn.	*Colomerus coplus* Manson, 1984
15’	Median line distally indistinct; opisthosoma with 48–50 microtuberculate annuli; microtubercles fading dorsally on posterior 10 annuli; opisthosomal setae *d* and *e* 36 and 40 µm, respectively; on *Vitex wilmsii*	*Colomerus vitexi* Meyer & Ueckermann, 1990
16	Without evident ocellar gibbosities; opisthosoma with 50–57 microtuberculate annuli; microtubercles rectangular dorsally, fading on posterior 10 annuli; on *Antidesma venosum*	*Colomerus antidesmae* Meyer & Ueckermann, 1990
16’	With or without evident ocellar gibbosities; opisthosoma with 50–59 microtuberculate annuli; microtubercles oval dorsally, may be missing on posterior-most annuli; on other hosts	17
17	Frontal lobe of prodorsal shield much broader than long; with ocellar gibbosities (sometimes not well distinct); opisthosoma with 54–59 microtuberculate annuli; microtubercles fading dorsally on posterior 15 annuli; on *Tinnea barbata*	*Colomerus tinneae* Meyer & Ueckermann, 1990
17’	Frontal lobe of prodorsal shield about as broad as long or slightly broader than long; with or without evident ocellar gibbosities; opisthosoma with 50–55 microtuberculate annuli; posterior-most opisthosomal dorsal annuli with or without microtubercles; on other hosts	18
18	Region between admedian lines on prodorsal shield with many short lines; with prominent ocellar gibbosities; opisthosoma with 55 microtuberculate annuli; on *Alangium saviifolium*	*Colomerus alangii* Keifer, 1978
18’	Region between admedian lines on prodorsal shield only with median line; without prominent ocellar gibbosities; opisthosoma with 50–55 microtuberculate annuli; posterior dorsal 15 annuli without microtubercles; on *Ziziphus mucronata*	*Colomerus mansus* Meyer & Ueckermann, 1990
19	Opisthosoma with 70–94 annuli; with evident ocellar gibbosities	20
19’	Opisthosoma with at most 66 annuli; with or without evident ocellar gibbosities	21
20	Opisthosomal setae *d* and *e* 40–46 and 38–60 µm, respectively; opisthosoma with 76–89 microtuberculate annuli; posterior 6 dorsal annuli sparsely microtuberculate (all microtuberculate according to Mathez 1965 and Newkirk and Keifer 1971); on *Vitis vinifera*	*Colomerus vitis* (Pagenstecher, 1857)
20’	Opisthosomal setae *d* and *e* 31 and 27 µm, respectively; opisthosoma with 75–94 microtubertulate annuli; on *Ribes nigrum*	*Colomerus riberini* Shi & Boczek, 2002
21	Ocellar gibbosities absent; genital coverflap with longitudinal ridges arranged in a single row	*Colomerus lepidaturi* (Farkas, 1960)
21’	Ocellar gibbosities well evident, ill-defined or absent; genital coverflap with longitudinal ridges arranged in two transverse rows	22
22	With evident ocellar gibbosities; opisthosoma with about 62 microtuberculate annuli; microtubercles broadly oval; on *Spathodea campanulata*	*Colomerus spathodeae* (Carmona, 1967)
22’	With ill defined ocellar gibbosities; opisthosoma with 49–61 microtuberculate annuli, of which the 8–10 posterior-most without microtubercles; microtubercles elongate dorsally and ventrally, shorter and more rounded laterally; on *Terminalia* (syn. *Buchenavia*, *Bucida*) *buceras*	*Colomerus bucidae* (Nalepa, 1904)

### Genera close to *Colomerus*

*Ectomerus* Newkirk & Keifer is the genus that most closely resembles *Colomerus* morphologically. It was described as a monotypic genus by Newkirk and Keifer (1975) in a dichotomous key to the genera of Cecidophyinae, with *Stenacis anysis* Keifer as the type species, described by Keifer (1970). Presently, three other species (*Ectomerus chebulae* Mohanasundaram, 1980; *Ectomerus systenus* Meyer, 1990; *Ectomerus triquetrus* Flechtmann & Etienne, 2002) are also included in this genus (Amrine and Stasny 1994, Amrine and de Lillo 2013 pers. comm.). The main characteristic used by Newkirk & Keifer to separate *Ectomerus* from *Colomerus* was its narrow and “basally flexible” anterior lobe; the flexibility of the anterior lobe was probably assumed by the observed variability of the angle between the lobe and the gnathosoma in lateral view of mounted specimens, although the authors also state seta *h1* to be present (though minute) and female genitalia not to be strongly appressed to the coxisternum II.

*Palmiphytoptus* Navia & Flechtmann is also similar to this genus. It was described (Navia and Flechtmann 2002) based only on the type species, *Palmiphytoptus oculatus* Navia & Flechtmann, 2002. This genus was described in Phytoptidae. Amrine et al. (2003) suggested the possibility that these mites could belong to Eriophyidae (probably *Eriophyes*), assuming the possibility that the setae interpreted as *ve*, could refer to *sc*, located much anterior to their usual position. *Palmiphytoptus barbosae* Navia & Flechtmann was described more recently (Navia and Flechtmann 2005). The genital apodemes of species of this genus seem similar to that of *Colomerus* and although in the type species the coverflap is not appressed to the coxisternum II, in *Palmiphytoptus barbosae* it is appressed. We consider that regardless of the placement of this genus at the family and subfamily level, the placement of the prodorsal shield setae would make it different from *Colomerus*.

### Economic importance of *Colomerus*

The kinds of injury caused by *Colomerus* species are very diverse, with some species causing more than one type of damage. The main types of damage are mentioned as: disturbance to development of new leaves, by damaging buds (*Colomerus oculivitis*, *Colomerus vitis*, *Colomerus woodfordis*), fruit deformation (*Colomerus bucidae*), formation of leaf erinea (*Colomerus alangii*, *Colomerus bucidae*, *Colomerus coplus*, *Colomerus holodisci*, *Colomerus mespiliformae*, *Colomerus nudi*, *Colomerus riberini*, *Colomerus spathodeae*, *Colomerus tricaseri*, *Colomerus vitexi*, *Colomerus vitis*, *Colomerus volkensiae*), leaf outgrowth (*Colomerus tricaseri*), “witch’s broom”, by damaging inflorescences (*Colomerus antidesmae*), formation of leaf galls (*Colomerus bucidae*, *Colomerus lepidaturi*, *Colomerus neopiperis*, *Colomerus tinneae*, *Colomerus trichodesmae*), leaf distortion (*Colomerus mansus*, *Colomerus spathodeae*, *Colomerus trichodesmae*, *Colomerus vitis*) and fruit necrosis (*Colomerus novahebridensis*). The following species were not associated with any type of damage on plants from which type specimens were collected: *Colomerus codiaeum* and *Colomerus gardeniella*.

While several of these species are known to attack ornamental plants, only 3 species have been reported from major crops: *Colomerus oculivitis* and *Colomerus vitis* from grapevine and *Colomerus novahebridensis* from coconut. *Colomerus oculivitis* and *Colomerus vitis* have been mentioned to cause economic damage to their host, especially *Colomerus vitis*, which has a wide distribution (Jeppson et al. 1975; Duso and De Lillo 1996; Craemer and Saccaggi 2013). *Colomerus novahebridensis* is usually not considered a pest, although West African cultivars growing in the Philippines and Malaysia are mentioned to be sometimes significantly damaged (Howard et al. 2001). As reported previously in this paper, this species was usually found in this study at very low levels, causing little damage; in a few occasions, damage was slightly more extensive, and the mite apparently caused premature fruit drop (see further details under “relation to host” in the complementary of the species based on the Thai population).

## Supplementary Material

XML Treatment for Colomerus novahebridensis
